# Catecholamine concentrations in duck eggs are impacted by hen exposure to heat stress

**DOI:** 10.3389/fphys.2023.1122414

**Published:** 2023-02-06

**Authors:** Joshua M. Lyte, Mark Lyte, Karrie M. Daniels, Esther M. Oluwagbenga, Gregory S. Fraley

**Affiliations:** ^1^ Poultry Production and Product Safety Research Unit, Agricultural Research Service, United States Department of Agriculture, Fayetteville, AR, United States; ^2^ Department of Veterinary Microbiology and Preventive Medicine, College of Veterinary Medicine, Iowa State University, Ames, IA, United States; ^3^ Department of Animal Sciences, Purdue University, West Lafayette, IN, United States

**Keywords:** norepinephrine, microbial endocrinology, avian, heat stress, duck, egg, catecholamine

## Abstract

Rapid “fight-or-flight” responses to stress are largely orchestrated by the catecholamines. Moreover, catecholamines and catecholamine precursors are widely recognized to act as interkingdom signaling molecules among host and microbiota, as well as to serve as chemotactic signals for bacterial foodborne pathogens. While albumen and yolk concentrations of glucocorticoids have received extensive attention as non-invasive indicators of hen response to stress, little is known regarding the impact of the hen’s stress response on *in ovo* catecholamine and catecholamine precursor concentrations. The aim of the present study was to determine norepinephrine and L-dopa concentrations in albumen and yolk of eggs laid by hens maintained under normal or heat stress conditions. Norepinephrine and L-dopa concentrations were also measured in oviductal tissue. Breeder ducks (∼35 weeks/age) were kept under normal (22°C) conditions or subjected to cyclical heat stress (35°C day/29.5°C night) for 3 weeks. Eggs (*n* = 12 per timepoint/group) were collected on a weekly basis. Hens were sacrificed at baseline or after 3 weeks of heat stress for oviductal tissue collection. Albumen, yolk, and oviduct concentrations of norepinephrine and L-dopa were determined using ultra high-performance liquid chromatography with electrochemical detection. Norepinephrine and L-dopa were detected in oviductal tissue as well as egg albumen and yolk. Norepinephrine concentrations were elevated (*p* < 0.05) in the yolk of eggs laid by the heat stress group compared to those of the control group. Norepinephrine concentrations in albumen were elevated (*p* < 0.05) in the heat stress group compared to control group at week 2. L-dopa concentrations were not significantly affected (*p* > 0.05) by heat stress in albumen, yolk, or oviductal tissue. Together, the present study provides the first evidence of the stress neurohormone, norepinephrine, in duck eggs and identifies that hen exposure to heat stress can affect *in ovo* norepinephrine concentrations. These data highlight the potential utility of *in ovo* catecholamine concentrations as non-invasive measures of the hen’s response to stress, as well as warrants future research into whether hen deposition of stress-related neurochemicals into the egg could serve as a chemotactic signal in the vertical transmission of foodborne pathogens.

## Introduction

Stress is a critical consideration in all aspects of poultry production (Chen et al., 2021) and is also an important factor in *Salmonella* spp. control in poultry flocks (Humphrey, 2006). As such it should not be surprising that animal welfare is a major determinant in shaping disease susceptibility and influencing foodborne pathogen carriage in birds. Avian, as well as mammalian, neuroendocrine responses to stress involve the production of stress-related neurochemicals that are released into circulation, the gut lumen and other peripheral sites (Villageliu and Lyte, 2017), as well as deposited into yolk and albumen contents during egg formation (Moudgal et al., 1990b). While having impact on host physiology, stress-related neurochemicals, such as norepinephrine, have been recognized for decades to act as interkingdom signaling molecules, including to elicit an increase in bacterial growth and colonization as well as to upregulate virulence in the host both *in vitro* and *in vivo* (Lyte et al., 2022).

A thorough understanding how heat stress can affect norepinephrine concentrations in egg albumen and yolk can serve two complementary outcomes for poultry producers. First, measuring catecholamine concentrations, including norepinephrine and l-3,4-dihydroxyphenylalanine (L-dopa), in egg contents can serve as a novel non-invasive measure of hen’s response to stress (Moudgal et al., 1990a). Second, norepinephrine concentrations can inform the design of a potential microbial endocrinology-based strategy for the control of vertical transmission of *Salmonella* spp. to prevent bacterial migration from an infected hen oviduct into the albumen and yolk (Methner et al., 2008). Indeed, stress-related neurochemicals, including the catecholamine norepinephrine, can exert a chemotactic effect on bacteria (Lopes and Sourjik, 2018). Hence it can be hypothesized that increased norepinephrine concentrations in egg contents due to hen’s stress response could provide a chemotactic signal for the migration of *Salmonella* spp. into the egg contents. Nevertheless, very limited attention has been directed towards the impact of stressors in modern poultry production, including the climate change challenge of heat stress (Lara and Rostagno, 2013), on hen deposition of catecholamines in egg contents or in the oviduct tract. Therefore, we sought to determine whether heat stress can increase norepinephrine and L-dopa concentrations in egg albumen and yolk, as well as oviductal tissue of healthy breeder ducks. The results of this study provide poultry researchers with the foundation to explore whether egg neurochemical concentrations could be used as a non-invasive measure of hen’s stress response and are detectable at relevant concentrations to plausibly serve as a chemotactic attractant for vertical transmission of *Salmonella* spp.

## Materials and methods

### Ducks and egg collection

None of the breeder ducks in this experiment had any prior exposure to heat stress (HS). The ducks were placed in single rooms with an 18:6 light cycle, temperature of 20–22°C for both treatment groups until 85% lay (∼35 weeks of age). Water nipple lines (5 ducks per nipple) were placed over a pit covered with raised plastic flooring, and the remaining area of the rooms were covered with pine shavings and added to or replaced as necessary at the same time for both rooms. Nest boxes were placed along one wall of the room with four hens per nest box as per industry standards (Chen et al., 2021), and all eggs collected daily. Details of the study design were published elsewhere (Oluwagbenga et al., 2022). Briefly, heat stress or control conditions began when the hens reached 85% lay. The HS group was subjected to cyclic temperature of 35°C for 10 h/day and returned to 29.5°C for the remaining 14 h/day for 3 weeks while the control ducks were housed at an industry-standard temperature of 22°C. These temperature ranges were selected as being typical temperatures during periods of heat stress in the midwestern United States and to be coincident to decreases in fertility observed in commercial duck barns. All procedures and management practices were approved by Purdue University Institutional Animal Care and Use Committee (Protocol #2109002195) prior to the start of the study.

Eggs from control and heat stress groups (*n* = 12 eggs/group/timepoint) were collected on the same day prior to the onset of HS (baseline), and then at the end of each week over the 3 weeks heat stress period. Details of the egg collection procedure were previously published (Oluwagbenga et al., 2022). Due to the number of hens compared to number of nest boxes typical for industry and research, we are not capable at this time to associate an egg with the hen whom laid that specific egg. To collect oviductal tissue, breeder ducks (*n* = 9–10/group/timepoint) were euthanized (intravenous pentobarbital; FatalPlus, 396/mg/mL/kg) at baseline (i.e. before the start of heat stress) or at the end of the 3 weeks heat stress period for oviductal tissue collection. An *n* = 12 eggs per group/timepoint and an *n* = 9–10 ducks/group/timepoint for oviduct samples were used in the present study as these n numbers provided sufficient Power to detect a significant effect (*p* < 0.05) of stress on egg yolk stress-related neurochemical concentrations (Moudgal et al., 1990a; Moudgal et al., 1990b). After breaking the eggshell, the yolk and albumen were carefully separated. 900 μL of yolk or albumen were separately pipetted into individual 2 mL reinforced tubes each containing 100 μL of 2N perchloric acid, vortexed, then snap frozen on dry ice. Oviductal tissue was collected from the magnum region of the reproductive tract, weighed, and then acidified in individual 2 mL reinforced tubes containing 1 mL of 0.2N perchloric acid and six ceramic beads, then snap frozen on dry ice. All yolk, albumen, and oviduct samples were stored at −80°C until analysis.

### Ultra-high performance liquid chromatography with electrochemical detection

All samples were processed and analyzed as previously described (Lyte et al., 2022). Briefly, thawed samples were homogenized in a BeadRuptor and then centrifuged at 3,000 × g and 4°C for 15 min. Yolk samples required heating to 37°C and diluting 1:10 with mobile phase in order to pass through the spin filters. Sample supernatant was passed through 2–3 kDa spin filters, and flow-through was stored at −80°C until ultra-high performance liquid chromatography with electrochemical detection (UHPLC-ECD). The UHPLC-ECD consisted of a Dionex Ultimate 3,000 autosampler, a Dionex Ultimate 3,000 pump, and Dionex Ultimate 3,000 RS electrochemical detector (ThermoFisher-Scientific, Sunnyvale, CA). Mobile phase was buffered 10% acetonitrile (Catalog #: NC9777698, ThermoFisher-Scientific) and the flow rate was 0.6 mL/min on a 150 mm (length) 3 mm (internal diameter) 3 µm (particle size) Hypersil BDS C18 column (Catalog #: 28,103-153030, ThermoFisher-Scientific). A 6041RS glassy carbon electrode set at 400 mV was used for electrochemical detection. Data were analyzed using the Chromeleon software package (version 7.2, ThermoFisher-Scientific), and neurochemical identification was confirmed using the retention time of the corresponding analytical standard (Millipore-Sigma, St. Louis, MO) (for norepinephrine, Catalog #: 636-88-4; for L-dopa, Catalog #: 59-92-7).

### Statistical analysis

Grubbs’ test (alpha = 0.05) was used to identify outliers in each dataset (GraphPad Prism v9.4.1, San Diego, CA). Data were analyzed with outliers removed followed by two-way ANOVA with Šídák’s *post hoc* test (GraphPad Prism v9.4.1). Stress and time were considered as main effects. Differences were considered significant at the threshold of *p* < 0.05.

## Results

**FIGURE 1:**
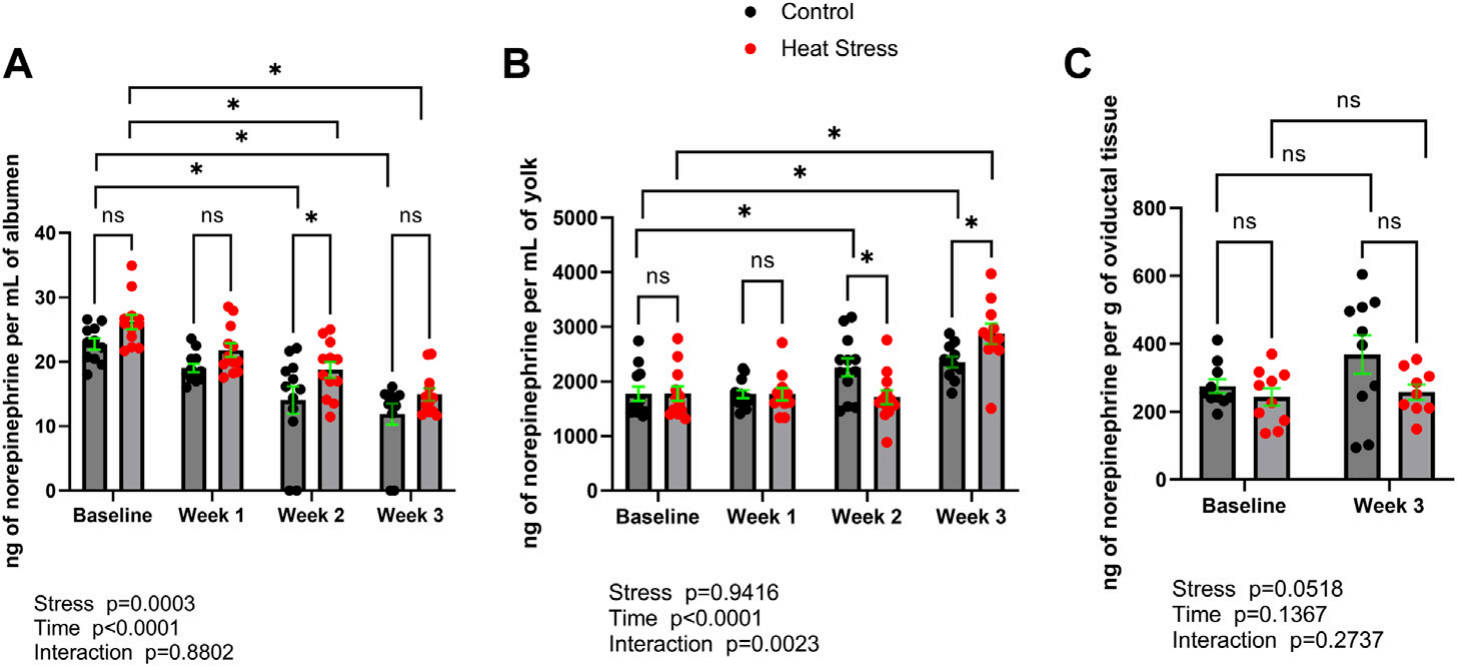
Norepinephrine concentrations in albumen **(A)**, yolk **(B)**, or oviductal tissue **(C)** in control or heat stress groups at baseline or after 1, 2, or 3 weeks of heat stress. Significant differences (*p* < 0.05) were indicated with *. Values are ng per mL of albumen or yolk, or ng per g of oviductal tissue. All values are expressed as mean ± SEM (*n* = 12 eggs/timepoint/group or for oviductal tissue *n* = 9–10 ducks/timepoint/group).

Norepinephrine (Figure 1) was detected in albumen, yolk, as well as oviductal tissue in both control and heat stress groups. Norepinephrine concentrations were not different (*p* > 0.05) between control and heat stress groups at baseline in albumen, yolk, or oviduct. No differences between control and heat stress groups were observed in norepinephrine concentrations in either albumen or yolk at week one. In albumen, norepinephrine was significantly elevated (*p* < 0.05) at week two in the HS group compared to the control. In yolk, norepinephrine was lower at week two relative to control, and significantly elevated (*p* < 0.05) compared to control at week three. There was a significant interaction for stress x time in yolk norepinephrine (*p* = 0.0023). Oviduct norepinephrine concentrations did not differ (*p* > 0.05) between control and HS groups at week three. Time-related changes were observed in both control and HS groups. In albumen, at weeks two and three norepinephrine concentrations were lower in both control and HS groups compared to baseline levels. In yolk, norepinephrine levels were higher at week two and three in control compared to baseline, and greater at week three in HS group compared to respective baseline samples. Differences between week three and baseline in oviduct norepinephrine concentrations were not observed (*p* > 0.05) for either control or HS groups.

**FIGURE 2:**
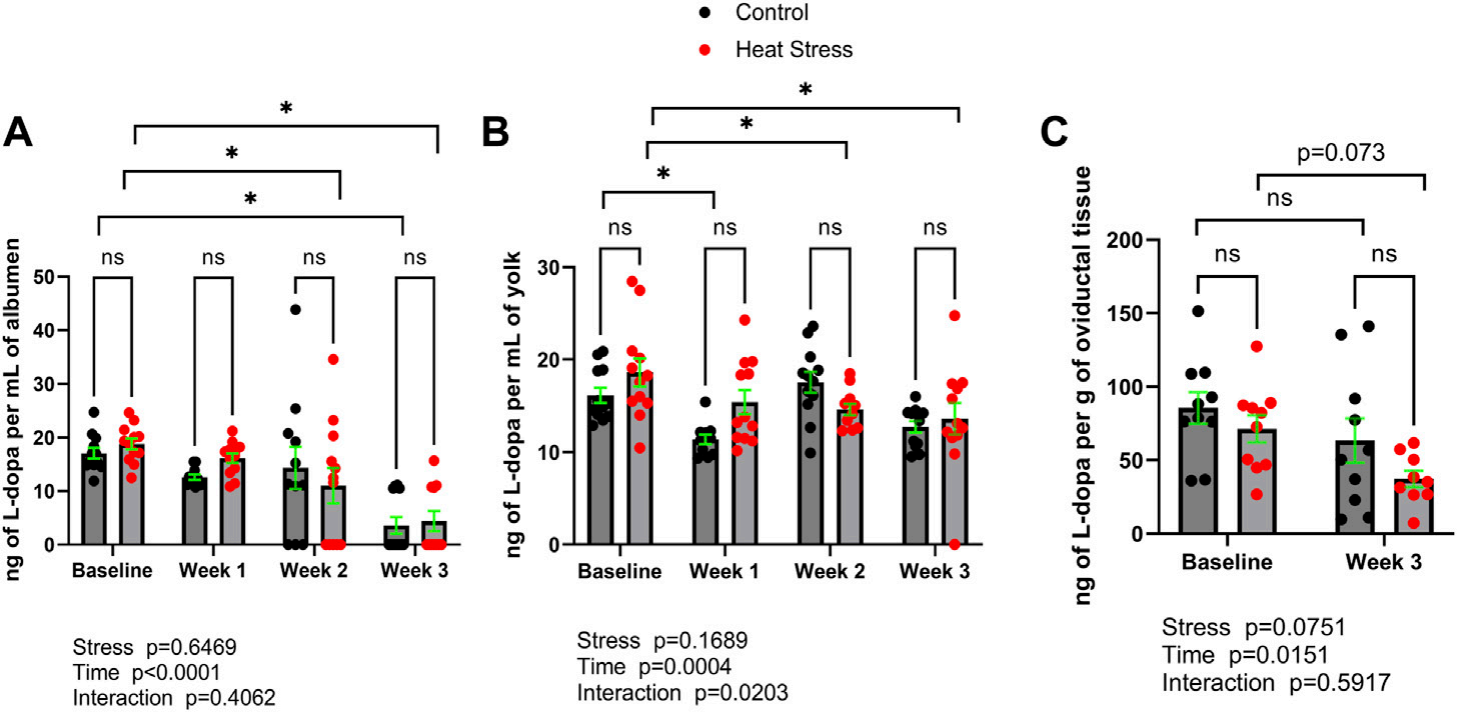
L-3,4-dihydroxyphenylalanine (L-dopa) concentrations in albumen **(A)**, yolk **(B)**, or oviductal tissue **(C)** in control or heat stress groups at baseline or after 1, 2, or 3 weeks of heat stress. Significant differences (*p* < 0.05) were indicated with *. Values are ng per mL of albumen or yolk, or ng per g of oviductal tissue. All values are expressed as mean ± SEM (*n* = 12 eggs/timepoint/group or for oviductal tissue *n* = 9–10 ducks/timepoint/group).

L-dopa concentrations are reported in Figure 2. L-dopa concentrations were not significantly different (*p* > 0.05) between control and HS groups at any timepoint in albumen, yolk, or oviduct. Time-related changes in L-dopa concentrations were found in both control and HS groups. In albumen, lower concentrations relative to baseline were found in the HS group at weeks two and three, while in control group only at week three compared to respective baseline group. In yolk, a decrease in L-dopa concentrations was observed (*p* < 0.05) in the HS group at weeks two and three compared to baseline. There was a significant interaction for stress x time in yolk L-dopa (*p* = 0.0203). Decreased oviduct L-dopa concentrations in week three relative to baseline groups were not statistically significant (*p* > 0.05).

## Discussion

While glucocorticoids are detectable in egg contents (Caulfield and Padula, 2020; Oluwagbenga et al., 2022), limited attention has been directed towards how stressors encountered in modern poultry production, such as heat stress, may affect catecholamine and catecholamine precursors in albumen and yolk. Previously it was reported that catecholamines are detectable in chicken egg yolk and are elevated in response to stress (Moudgal et al., 1990a; Moudgal et al., 1990). The present study is the first to demonstrate that catecholamines and catecholamine precursors, specifically norepinephrine and L-dopa, respectively, are also present in duck hen eggs and their levels are impacted by stress similarly to that observed in chickens (Moudgal et al., 1990b; Moudgal et al., 1990b).

The concentrations of norepinephrine reported here (Figure 1) are similar to those previously identified in chicken egg yolk (Moudgal et al., 1990a), indicating translational, or phylogenetic, utility of findings that describe neurochemical concentrations between ducks and chickens exposed to stress. Interestingly, when compared to the matching control group, we observed that norepinephrine concentrations in egg yolk of the heat stress group were lower and elevated at week two and week three, respectively. This result may suggest temporary adaptation of the hen’s stress response to heat stress. Indeed, previous reports in mammals have shown that chronic repeated forms of stress, including temperature based stressors such as cold exposure, cause a prolonged dampening of norepinephrine concentrations and other neuroendocrine components of the sympathetic stress response (Stone and McCarty, 1983; Konarska et al., 1990). As eggs were collected in the present study at the end of each week of heat stress, future studies should investigate whether at the beginning of the heat stress period (i.e. sampling eggs at the beginning of week one of heat stress) there may be an initial immediate stress response.

We also sought to quantify a precursor to norepinephrine synthesis, L-dopa, concentrations (Figure 2) in egg contents and oviduct. L-dopa is a precursor to norepinephrine and is able to be added to feed and has been reported to directly modulate catecholamine synthesis in stress-susceptible pigs *in vivo* (Erlander et al., 1985). While L-dopa was not found to be increased (*p* > 0.05) in albumen, yolk, or oviduct in response to stress, the fact that L-dopa was detectable in egg contents and the oviduct provides a possible platform for the design of in-feed manipulation of L-dopa, and therefore *in ovo* norepinephrine concentrations. This could be especially useful as a potential microbial endocrinology-based strategy to control the impact of stress on vertical transmission of *Salmonella* spp. via hen norepinephrine biosynthesis. It was previously shown that norepinephrine not only promotes *Salmonella* spp. colonization in chicken layers *in vivo*, but that in egg whites *in vitro* can enable *Salmonella* spp. growth (Methner et al., 2008). Moreover, as norepinephrine can exert chemotactic effects on bacteria (Lopes and Sourjik, 2018), *Salmonella* spp. can migrate from albumen to the yolk (Braun and Fehlhaber, 1995), it is reasonable to hypothesize that environmental stress that causes the hen to deposit norepinephrine in the egg contents could serve to encourage vertical transmission of *Salmonella* spp. from infected oviduct to the egg. Alternatively, stress hormones could be deposited into egg albumen as a way of transferring information about the environment to the embryo.

Avian stress hormones have been demonstrated to be transferred to egg contents, including the yolk (Hayward and Wingfield, 2004). Maternal hormones in the avian egg are means of transferring information from one generation to the other showing how the mother can contribute to the coping mechanism of the offspring to environmental variability (von Engelhardt and Groothuis, 2011). Glucocorticoids, androgens, and nutrients can be transferred from mother to egg via the yolk and albumen and can have significant effects on the phenotype of the developing offspring (Eising and Groothuis, 2003; Hayward and Wingfield, 2004; Caulfield and Padula, 2020). In the same eggs analyzed in this study, we separately assessed glucocorticoid levels and found increased cortisol in the albumen (Oluwagbenga et al., 2022). The combination of both catecholamine and glucocorticoid may lead to specific patterns of DNA methylation during embryogenesis, although this has yet to be determined and requires further studies. However, several studies have elevated yolk androgen concentrations experimentally to assess the effects on the developing offspring. These effects include earlier hatching time in black-headed gull (Eising and Groothuis, 2003), increased muscular development in red-winged blackbird (Lipar and Ketterson, 2000), enhanced post-hatching growth in canaries (Schwabl, 1996), more intense food solicitation behavior in black-headed gulls (Eising and Groothuis, 2003), improved nestling survival in zebra finches (von Engelhardt et al., 2006), and decreased early immune function in Chinese painted quail (Andersson et al., 2004). Thus, there is ample evidence for elevated hormone levels in egg to alter offspring phenotypes.

Together, the present study establishes the presence of norepinephrine and L-dopa in duck egg contents, and that hen exposure to heat stress can alter catecholamine concentrations in both albumen and yolk.

## Data Availability

The raw data supporting the conclusions of this article will be made available by the authors, without undue reservation.
